# Myeloid Colony-Stimulating Factors as Regulators of Macrophage Polarization

**DOI:** 10.3389/fimmu.2014.00554

**Published:** 2014-11-21

**Authors:** Thomas A. Hamilton, Chenyang Zhao, Paul G. Pavicic, Shyamasree Datta

**Affiliations:** ^1^Department of Immunology, Lerner Research Institute, Cleveland Clinic Foundation, Cleveland, OH, USA

**Keywords:** macrophage activation, macrophage colony-stimulating factor, granulocyte macrophage colony-stimulating factor, inflammation, cytokines

## Abstract

The scope of functional heterogeneity in macrophages has been defined by two polarized end states known as M1 and M2, which exhibit the proinflammatory activities necessary for host defense and the tissue repair activities required for restoration of homeostasis, respectively. Macrophage populations in different tissue locations exist in distinct phenotypic states across this M1/M2 spectrum and the development and abundance of individual subsets result from the local and systemic action of myeloid colony-stimulating factors (CSFs) including M-CSF and GM-CSF. These factors have relatively non-overlapping roles in the differentiation and maintenance of specific macrophage subsets. Furthermore, there is now evidence that CSFs may also regulate macrophage phenotype during challenge. Cell culture studies from multiple laboratories demonstrate that macrophages developed in the presence of GM-CSF exhibit amplified response to M1 polarizing stimuli while M-CSF potentiates responses to M2 stimuli. As a consequence, these factors can be important determinants of the magnitude and duration of both acute and chronic inflammatory pathology and may, therefore, be potential targets for therapeutic manipulation in specific human disease settings.

## The Macrophage Polarization Paradigm: Classical (M1) and Alternative (M2) Activation Phenotypes

The mononuclear phagocyte system is known to exhibit substantial functional heterogeneity (1–3). Though the scope of such heterogeneity is dramatically large, the prevailing concept of heterogeneity is organized around two polarized endpoints known as classical and alternative activation, also often termed M1 and M2, respectively (4–6). These polarized states represent the capacities to initiate inflammatory response, carry out anti-microbial function, and promote Th1/Th17 adaptive immune responses for M1, or, for M2, to phagocytize debris, promote wound healing, antagonize destructive inflammation, and suppress adaptive immunity. These functional activities are mediated by molecular features that include, for M1, the production of proinflammatory cytokines, co-stimulatory molecules including CD80/86, effector enzymes such as iNOS, and NADPH oxidase or, for M2, anti-inflammatory cytokines, immunosuppressive arginase, and scavenger receptors (CD163) (5, 6).

Importantly, there are multiple subsets of macrophages with relatively non-overlapping functional responsibilities and this additional heterogeneity must be superimposed upon the M1/M2 paradigm (2, 7–9). Resident macrophage populations exhibit many common features but are not identical, often deriving from distinct origins and showing a broad spectrum of functional potential (10). It is noteworthy that most resident tissue macrophages are capable of self-replenishment and generally do not derive from circulating monocytes (9, 11). There are two major categories of monocytes found in the circulation. In the mouse, these are characterized as Ly6C-hi/CCR2^hi^ and Ly6C^lo^/CX3CR1^hi^. The former is likely the source of most infiltrating inflammatory macrophages or dendritic cells, while the latter is a patrolling and longer lived cell population that provides maintenance of the vascular endothelium (7). The understanding of macrophage subsets and their developmental origin has advanced dramatically through the application of fate mapping and other transgenic strategies (2, 11–13). Moreover, the molecular features responsible for distinct functional activities (anti-microbial, inflammatory, reparative, etc.) have been defined through detailed analysis of isolated cell populations and cell type-specific transgenic manipulation (5, 6, 14). These macrophage subpopulations exhibit many molecular similarities that reflect their common myeloid origin but each has very distinct responsibilities and their inter-conversion appears to be limited under steady-state conditions (9). Moreover, each of these subset populations can be induced to exhibit M1 or M2 like functional polarization.

The concepts of classical and alternative activation or even M1 and M2 markedly oversimplify the spectrum of macrophage phenotypes that exist within vertebrate organisms (6, 15). The number of individual phenotypes will, in part, depend upon the number of gene products that are measured and the degree to which gene expression events are independent (16). Furthermore, the spectrum of environmental cues encountered by macrophages is highly complex both in number and exposure sequence. Hence, the combinatorial spectrum of possible phenotypes is extremely large. As a simplifying principle, the number of specific molecular endpoints used to define phenotype can be limited to those activities and molecules requisite to the functions of interest (e.g., anti-microbial, reparative, etc.). This will enable comparison of macrophage populations within different physiologic and pathologic circumstances and evaluation of how the specific molecular characteristics may vary with cell subset and stimulus environment.

Finally, it is also important to recognize that inflammation is a dynamic process that is well recognized to proceed in stages with an early proinflammatory function followed by a transition to healing and restoration of tissue homeostasis (1, 17, 18). While this process includes the temporally distinct recruitment of multiple cell populations that can provide different aspects of the evolving functional features, there is also reason to believe that infiltrating inflammatory monocytes may cycle through different states of functional activity that are, at a minimum, reminiscent of the M1 and M2 categories (19, 20).

## Regulation of Polarization

Classically and alternatively activated (M1 or M2) macrophage phenotypes are defined by the specific molecular characteristics induced in response to prototypic pro- and anti-inflammatory stimuli [e.g., IFNγ, Toll-like receptor (TLR) agonists, IL-4/IL-13, IL-10, etc.] (5, 15, 18). Such molecular correlates are now used with increasing frequency to identify populations of M1 or M2 like macrophages in different physiologic settings. Thus, M1 macrophages express high levels of IL-12 or IL-23, TNFα, IL-1α/β, chemokines eliciting neutrophil, inflammatory monocyte, and proinflammatory T lymphocyte infiltration (CXCL1-3, 8, CCL2, CXCL9, and CXCL10), CD80/86, CD64, MARCO, iNOS, and reactive oxygen species (hydroxyl radicals and H_2_O_2_) induced by M1 polarizing stimuli. Correlates of the M2 phenotype include IL-10, TGFβ, arginase, YM1, FIZZ1 (in the mouse), the mannose receptor (CD206), and scavenger receptors, such as CD163 induced following exposure to IL-4, IL-13, TGFβ, IL-10, and other agents.

In this context, M1 and M2 phenotypes are often presented as relating to the actions of Th1 and Th2 cell subsets, respectively, because of the Th1 and Th2 lymphokine-based modulation of their defining features (15, 21). It is evident, however, that polarized macrophage function existed prior to the development of adaptive immunity in evolution (22, 23). Moreover, mice with T- and B-cell deficiency still possess the potential for polarized function demonstrating that the M1 and M2 concept can exist in the absence of adaptive immunity and its products (4). Nevertheless, the normal vertebrate immune system operates with a full spectrum of immune cell populations and their products clearly influence the macrophage polarization process. Indeed, it is likely that macrophages will encounter both M1 and M2 polarizing stimuli simultaneously within the inflamed tissue microenvironment. The complexity of response to this may reasonably explain the large spectrum of macrophage phenotypes encountered in cell populations *in vivo* and the variability in markers of polarization.

Macrophage populations may also exhibit a predisposition for polarization toward the M1 or M2 phenotype. For example, different strains of mice and rats have been shown to have skewed patterns of activation potential that ultimately correlate with their innate and adaptive immune functionality (4, 24, 25). Furthermore, it is well recognized that macrophages in different anatomic or physiologic settings exhibit dramatically different capacities for polarization. Importantly, there are agents that, by themselves, do not induce expression of common polarization markers but which can alter response to stimulation with classical M1 or M2 stimuli. Because most, if not all macrophage populations, can respond to either M1 or M2 stimuli, the tone of a response may be set by differential regulation of sensitivity to polarizing stimuli. Hence, agents that promote priming of macrophages for enhanced or diminished response to classical or alternative activation are likely to be important determinants of the character and temporal patterns of macrophage functional change in the course of response to injury and infection.

The myeloid colony-stimulating factors (CSFs) M-CSF and GM-CSF are known to modulate macrophage phenotype (26, 27). While both agents were first identified as inducers of myeloid cell differentiation and proliferation in cultured bone marrow progenitors, many studies illustrate their importance in the magnitude, duration, and character of many forms of inflammatory response (26–30). Though GM-CSF is associated with classical or M1 activation while M-CSF is linked with alternative or M2 activation, neither factor is a potent stimulus of definitive polarization markers, when compared with prototypic polarizing stimuli (e.g., IFNγ, TLRs, IL-4, IL-10, etc.) (31–33). Instead, GM-CSF and M-CSF appear to induce a state in which macrophages are primed for M1 and M2 endpoints, respectively.

## Molecular and Cellular Phenotypes Produced by M-CSF and GM-CSF *In vitro*

While receptor signaling mechanisms mediating responses to M-CSF and GM-CSF are appreciated in basic detail (5, 6, 26), understanding of how these agents modulate functional polarization remains obscure. The potential for CSFs to regulate the responses of mature myeloid cell populations is well recognized. For example, the capacity of GM-CSF, but not M-CSF, to generate DCs in culture clearly demonstrated distinct functional roles (34). Verreck et al. initially demonstrated that GM-CSF or M-CSF treatment of myeloid cells in culture was able to selectively alter the magnitude of M1 or M2 polarized phenotypes following appropriate stimulation (31, 32). While GM-CSF cultured macrophages stimulated with LPS ± IFNγ produced large amounts of IL-23 or IL-12 and little IL-10, M-CSF cultured macrophages were unable to generate either IL-12 or IL-23 but did produce significant amounts of IL-10 under the same conditions. GM-CSF-treated cells produced appreciably higher levels of other proinflammatory cytokines including TNF, IL-18, IL-1β, and IL-6 in comparison to those grown in M-CSF. Furthermore, M-CSF-treated cells were more phagocytic but less competent in antigen presentation when compared with GM-CSF treated cells. For the most part, the growth factor-treated cells did not exhibit the full M1 or M2 phenotypes in otherwise unstimulated state but rather showed polarized sensitivity for corresponding response to IFNγ/TLR signaling. These findings led the authors to conclude that GM-CSF and M-CSF were promoting the development of monocyte-macrophages predisposed to exhibit differential M1 and M2 phenotypes, respectively. These findings were confirmed and extended by Fleetwood et al. using bone marrow derived macrophages from mice cultured in either GM-CSF or M-CSF (33). Interestingly, though GM-CSF is frequently used to generate DCs from bone marrow progenitors, the cells arising from such cultures more closely resemble macrophages than dendritic cells based upon whole genome profiling (35). Though there are many similarities between human being and mouse macrophages in different polarized states, there also appear to be many differences (14, 35–37). Despite these findings, there remain important questions about the strict relationship between these two factors and the molecular/functional phenotype definitions for M1 and M2. Thus, studies in both mouse and human cells show that the effects of GM-CSF and M-CSF on gene expression do not map exactly with M1 and M2 marker expression, even following stimulation (35, 38, 39).

In the context of the hypothesis that macrophage predisposition is an important determinant of polarized phenotype expression, there are certainly other natural ligands that have the capacity to alter macrophage sensitivity to specific polarization stimuli (40–45). Because inflammatory responses *in vivo* will always occur in a complex stimulus environment, these additional agents are likely to co-operate with or antagonize the actions of the CSFs. PPARγ, in particular, has been reported to be required for development of alternatively activated macrophages in the context of insulin resistance and metabolic inflammatory disease (46). The tyrosine kinase receptor CD136 (RON, MST1R) can also modulate sensitivity for M2-like activators, in part by altering the sensitivity to TLR stimulation (24, 47). In contrast to these agents, NOTCH and its ligand RBP-J, are reported to promote M1-like responses via alterations in intracellular signaling factors including IRF8 and SOCS3 (40). Indeed, the sensitivity of myeloid cell populations to polarizing stimuli appears to be controlled in part by alterations in the abundance or activity of the signaling pathway components that mediate responses to pro- and anti-inflammatory stimuli (41–44, 48–50). Thus, signaling adaptors, protein kinases, protein phosphatases, and transcription factors including members of the IRF, SOCS, Tec, and KLF families have all been implicated in controlling either M1 or M2 polarization. The mechanisms through which stimulus sensitivity is altered by ligand/receptor pairs, such as the CSFs, as well as others mentioned above remains to be fully elucidated but intracellular signaling factors are likely to be important targets.

## Role of M-CSF and GM-CSF in Macrophage Functional Polarization *In vivo*

M-CSF and GM-CSF have distinct effects on the development and expansion of myeloid cell populations in different anatomic settings (26, 27). Based upon studies of mice with targeted deletion of ligand and/or receptor genes, M-CSF is known to be required for the production and maintenance of many (though not all) tissue macrophage populations (51). In this regard, distinctions between receptor and ligand deficient mice revealed the existence of a second ligand (IL-34), which is now known to be necessary for the maintenance of a subset of tissue macrophage populations (microglia and Langerhans cells) (52). M-CSF and IL-34 function in homeostatic maintenance of tissue-resident macrophage populations through promoting viability and proliferation and both drive predisposition to M2 character (53). While M-CSF is found in the serum of healthy individuals and can be produced constitutively by epithelia, fibroblasts, endothelial cells, and by macrophages themselves, its expression can be elevated by inflammatory conditions in many cells including macrophages as well as T and B lymphocytes (27). In contrast to M-CSF or IL-34, GM-CSF deficiency has little impact on steady-state tissue macrophage populations with the exception of those found in the lung (26, 27). While GM-CSF is believed to be important for the development of infiltrating inflammatory DCs, recent findings show that M-CSF but not GM-CSF sensitivity is requisite for these cell populations (13). Importantly, GM-CSF, unlike M-CSF, is not detectable in most tissues at rest but expression is frequently induced during inflammatory or immune stimulation in many tissues and cell types (26, 27, 54).

While studies using animals with global or cell-type restricted deficiencies in M-CSF or GM-CSF ligand/receptor function do provide insight into their relative contributions to macrophage phenotypes during inflammatory responses *in vivo*, the interpretation of such experiments should be viewed with caution due to the impact of such deficiencies on development and/or abundance of specific myeloid subsets. These studies are effectively complemented by transiently manipulating M- or GM-CSF levels using specific ligand delivery or ligand/receptor antagonism. Results from multiple studies indicate that both M-CSF and GM-CSF can modulate the magnitude and character of inflammatory response in multiple tissue specific disease models (2, 26, 54–56). These include autoimmune encephalomyelitis (MS), atherosclerosis, arthritis, nephritis, lung inflammation, and cancer. In most cases, however, the mechanisms through which these endpoints are achieved have not been elucidated. Certainly, both factors have the capacity to promote survival and/or expansion of macrophage populations both systemically and in specific tissue locations and the decreased number of macrophages observed with CSF antagonism would likely result in reduced intensity and/or duration of disease (26). Multiple approaches including delivery of M-CSF, antibody-mediated depletion of the ligand, antibody-induced antagonism of receptor, or the use of receptor tyrosine kinase inhibitors have provided evidence supporting both positive and inhibitory roles for M-CSF in inflammatory diseases (26, 55). Interestingly, several recent reports demonstrate that antagonistic targeting of M-CSF can have appreciable benefit in tumor therapy as a consequence of altering the tumor-associated macrophage phenotype from M2 to M1 (49, 57–59).

There are several important considerations that can provide some speculative insight into the role for these CSFs in regulating the nature of macrophage polarization *in vivo*. First are the patterns of CSF expression within tissues both at rest and during inflammatory responses. While the expression of both factors can be amplified during response to various forms of tissue injury, it is apparent that M-CSF is produced constitutively in many tissues and is critical for the maintenance of resident tissue populations throughout the body. GM-CSF, in contrast, is rarely detectable except at times of injury and does not appear to be a critical determinant of macrophage numbers with a few exceptions (i.e., the lung). Second, resident macrophage populations are generally found to exhibit an M2-like phenotype under resting conditions, consistent with the need to minimize tissue damaging inflammatory reaction (10). This is certainly consistent with the ability of M-CSF to predispose toward an M2 phenotype in cell culture experiments involving both human monocytes and mouse bone marrow-derived macrophages. Third, GM-CSF has been shown to be a critical determinant of inflammatory injury in several model systems (60–62). Particularly, T-cell derived GM-CSF was recently shown to be critical for disease phenotype in experimental autoimmune encephalomyelitis, generated by IL-23 action on Th17 cells (60, 61). In this instance, GM-CSF selectively targeted infiltrating macrophages within the CNS. Finally, several studies examining responses to M-CSF and GM-CSF *in vitro* show that the M1/M2 phenotype can be reversibly modulated by GM-CSF/M-CSF exposure in cell culture and GM-CSF predisposition may be dominant (33, 39, 63) (Hamilton, unpublished). This is also supported by increased bioavailability (half-life) of GM-CSF, which might also contribute to its dominance relative to M-CSF. We would suggest then that M-CSF provides the default condition and will promote an M2 (healing) phenotype both at rest and in the absence of other forms of stimulation. Induced expression of GM-CSF (e.g., during adaptive T-cell driven immune responses) will provide the mechanism for retaining or re-expressing an M1 phenotype when conditions require (e.g., continued infection or injury). It is clear, however, that the specific effects of M-CSF and GM-CSF on macrophage polarization in cell culture models are unlikely to fully predict their effects *in vivo*. This reflects complexities associated with the variable nature of inflammatory injury, stimulus exposure, and distinct features of specific tissue microenvironments.

## Unanswered Questions

Macrophage heterogeneity or phenotype polarization is an area of high current interest and the impact of myeloid CSFs on this process *in vivo* is evident but poorly understood. Hence, we pose the following outstanding questions regarding the roles that M-CSF and GM-CSF may play, particularly through modulating sensitivity to M1 and M2 promoting stimuli with the expectation that answers will help to clarify the process and provide insights to therapeutic application. (1) What mechanisms are involved in skewing responses to polarizing stimuli? Can we identify specific CSF-induced patterns of gene expression that are requisite to generating macrophages predisposed for more potent responses to cytokines and pattern recognition receptors? Can we correlate outcomes obtained *in vitro* with those obtained *in vivo*? (2) What are the sources and timing of M-CSF and GM-CSF expression within specific tissues during different forms of inflammatory response? Which myeloid cell populations are the targets of the CSFs and how is this co-ordinated with the need to enhance or diminish specific aspects of function over the full course of inflammatory response? (3) Finally, we must begin to consider not only the mechanisms through which M-CSF and GM-CSF operate but also how these stimuli are integrated with the host of other agents encountered within inflammatory settings that also have marked influence on M1/M2 skewing?

## Conflict of Interest Statement

The authors declare that the research was conducted in the absence of any commercial or financial relationships that could be construed as a potential conflict of interest.
